# Supplementary material for: Are carbon emissions trading and green financial instruments synergistic? -Comprehensive quantitative research based on content analysis
Source: PLoS One. 2024 Mar 7;19(3):e0298601. doi: 10.1371/journal.pone.0298601 (PMC10919601; doi:10.1371/journal.pone.0298601)
Supplement: S1 Data — (ZIP) [file pone.0298601.s001.zip › Data/地级市Z_F工作报告.zip!/╜¡╬≈/╙Ñ╠╢╩╨/└┤╫╘╣1⁄2╓┌║┼í╛┬φ┐╦╩2╛▌═°í┐_2015.docx]

各位代表：

　　现在，我代表市人民政府，向大会作政府工作报告，请予审议，并请各位市政协委员和列席会议的同志提出意见。

　　一、2014年工作回顾

　　过去的一年，在省委、省政府和市委的正确领导下，全市上下全面贯彻“发展升级、小康提速、绿色崛起、实干兴赣”十六字方针，按照“主攻项目、决战‘三区’，凸现特色、实现跨越”总体要求，认真落实稳增长促改革调结构惠民生各项政策措施，全力推进“8+1”重点工作，经济社会保持了平稳健康较快发展的良好势头。预计（下同）全年完成生产总值606亿元，增长9.8%；财政总收入101.7亿元、增长9.7%，一般公共预算收入73.4亿元、增长11%；规模以上工业增加值340亿元，增长11%；固定资产投资455亿元，增长16%；社会消费品零售总额145亿元，增长12.6%；城镇居民人均可支配收入24300元、增长10%，农村居民人均可支配收入10920元、增长11%。

　　一年来，主要做了以下七个方面工作：

　　（一）改革创新力度进一步加大。围绕“小政府、大服务、活市场、优环境”，一系列关键领域的改革取得突破，进一步累积了发展升级的后发优势。政府机构改革稳步实施，整合组建市卫计委、市场监督管理局、机关事务管理局、农业和粮食局、白鹤湖现代农业示范区管理局、城市管理行政执法局和综合检验检测中心等机构，完成市政、环卫、园林绿化、公交公司等管理体制改革，建立“12345”政务服务热线。行政审批制度改革进展顺利，制定了市本级行政审批项目目录和行政职权目录，精简审批项目245项、精简率79.6%，清理归并行政权力760项、保留行政职权1248项；制定了工业服务业等企业注册登记联审联批和建设项目优化审批流程试行方案，联审联批系统上线运行。市场体制改革相继推出，制定了加快非公经济发展、公共领域扩大开放、政府向社会力量购买服务等文件，推进注册资本登记制度改革，新登记市场主体7600户，新登记资金25.42亿元，分别增长16.5%和12.3%，非公经济增加值占生产总值的比重达50%。财税金融体制改革扎实推进，全口径预算改革顺利实施，政府、部门和“三公”经费预决算全面向社会公开，综合治税平台上线运行；进一步优化市级四大融资平台，完成城市经营性国有资产集中统一授权经营，平台投融资规模和能力进一步增强。农村领域改革成效明显，基本完成农村土地承包经营权确权登记和集体土地所有权确权登记颁证工作，建立了市县乡村四级土地流转服务体系，土地流转面积30万亩、流转率达33%，全面完成国有林场改革。教育体制、文化体制、医药卫生体制等社会事业改革扎实推进。

　　（二）产业转型升级进一步加速。面对宏观经济下行压力加大的严峻考验，克服了铜产业震荡波动、物流运输业税收回落、房地产业持续走低等诸多困难，科学应对，主动作为，在调结构转方式促转型上取得明显成效，三次产业结构调整为7.9：62.4：29.7。工业经济加快发展。沿320国道产业集聚升级带建设稳步推进，全市规模以上工业主营业务收入达3300亿元、增长11.4%，市属工业增加值占比首次突破50%，工业对经济增长的贡献率达67.5%，我市列入国家工业绿色转型发展试点城市和国家第二批资源综合利用“双百工程”示范基地。铜产业取得新突破，内陆首个铜期货交割仓库和中江国际商品交易中心成功获批，铜精深加工业逆势上扬，淘汰落后产能22.75万吨，置换先进产能30万吨，铜合金材料列入全省20个工业示范产业集群。地方特色产业不断壮大，三川水工产业园、节能照明基地、硫磷化工基地分别进驻企业15家、18家、10家，水工产业列入全省60个工业重点产业集群，微型元件产业列入省级产业基地。旅游产业快速发展。成功承办第三届国际道教论坛，来自27个国家2500余名嘉宾参会，进一步提升了鹰潭在国内外的知名度和美誉度。十大旅游项目有序实施，“寻梦龙虎山”实景演出顺利试演，逍遥城主体完工，全省旅游重点产业集群现场推进会在我市召开，龙虎山景区被评为2014“美丽中国”十佳旅游景区，智慧龙虎山被评为中国智慧旅游创新项目。全市接待国内游客增长36.7%、旅游总收入增长43%，龙虎山景区接待游客增长34%、旅游直接收入增长24%。商贸物流业稳步发展。鹰南现代物流园周边环境整治成效明显，鹰西国际商贸物流园初显雏形，十大专业市场建设有序推进，胜华机电五金商贸城一期基本完工，手拉手汽车城、浙商联盟建材家居城、国际眼镜城投入试运营。上海钢联等电商平台投入使用，我市列入全国中小商贸流通企业服务体系建设试点城市。货运业实现销售收入67.49亿元、上缴增值税5.8亿元。现代农业集约发展。全年粮食总产达69.7万吨，实现“十一连增”。雷溪、杨溪现代农业核心示范区建设进展顺利，新增高标准农田12万亩，白鹤湖现代农业示范区获批国家级示范区。全市新增农民专业合作社410家、家庭农场120家、专业种养大户897家，183家市级以上农业龙头企业实现销售收入72亿元。

　　（三）新型城镇化步伐进一步加快。市域总体规划（2014—2030）编制完成中期成果，获批国家新型城镇化综合试点城市和省级“多规合一”试点市，城镇化率达54%，提高1.27个百分点。信江新区30平方公里内的主干路网基本贯通，建成区面积达8平方公里。老城区盐业公司、老一中、上下桂等棚户区和城中村改造有序推进，全市完成棚改面积188万平方米。高铁站前广场、市民广场、四海路延伸、龙虎山南大道改造和北极阁主体等项目全面竣工。贵溪东城区、余江城东新区、月湖新城建设快速推进，同城发展步伐不断加速。十强中心镇建设稳步实施，194个秀美乡村建设点全面完成，“一江两河”综合治理稳步推进，86座小(二)型以上水库除险加固全面竣工，完成人工造林4.7万亩，改造农村公路353.5公里，农村建房“三清理一整治”行动初见成效。城乡环境明显改观，农村环境综合整治和生态保护建设力度加大，节能减排完成年度目标任务，建成空气质量自动监测站，信江鹰潭段监测断面达标率100%，中小型水库水质专项治理取得较好成效，贵冶周边土壤修复、贵电脱硫脱硝改造等治理工程顺利完成。

　　（四）开放带动效应进一步凸显。全年引进省外5000万元以上项目资金240亿元、增长15.3%，实际利用外资2.15亿美元、增长11.9%，外贸出口8.8亿美元，连续五年被评为全国外贸百强城市。赣港、赣台经贸合作等重大招商活动成果丰硕，引进中船重工、养元“六个核桃”等5亿元以上项目14个，科明铜业、九隆科技等27个亿元以上项目建成投产，鑫发实业、昇兴包装等40个亿元以上项目开工建设。开放平台不断提升，海关、国检挂牌运营并列入全省关检“三个一”试点，电子口岸基本建成，鹰潭至宁波“五定班列”运行平稳。

　　（五）民生幸福指数进一步提升。十大惠民工程64件实事基本完成，民生支出占财政总支出的比重达63.4%，提高5.1个百分点。全市城镇新增就业2.78万人、城镇就业率96.8%，新增转移农村劳动力2.28万人。全省社会保险“多险合一”信息系统在我市试点运行，实现“五险”统一征缴、市内就医“一卡通”和异地就医即时结算。连续10年为企业退休人员提高养老金，养老金支付率和社会化发放率均达100%。全市近3万名被征地农民纳入城镇职工社会养老保险。启动农村居民大病保险试点，新农合人均筹资标准提高到390元。社会救助水平逐步提高，为困难群众发放生活补助资金2.68亿元。新开工建设公租房4000套，基本建成2512套。完成农村危房改造1657户。市区与余江供水管道连通应急用水工程顺利竣工。

　　（六）协调发展水平进一步提高。科技进步综合水平明显提升，省级创新研发平台达10个、省级高新技术产业基地达2个，新增高新技术企业实现翻番达32家，专利申请受理量和授权量实现年度双翻番，鹰潭高新区列入全省首批10个知识产权试点园区。鹰潭职院迁建工程进展顺利，城区学校改扩建、农村义务教育学校标准化建设有序推进，“大班额”治理成效明显。我市列为全省唯一一个居民健康卡发放试点城市，市人民医院信江新院主体完工，新建村级卫生计生服务室118个。“单独两孩”政策顺利实施。承办了中国文联“送欢乐、下基层”文艺志愿服务活动，举办了第七届海峡两岸道文化艺术交流论坛，首次晋京举办鹰潭历史文化之光展，黄蜡石文化集聚区正式开园。参加省第十四届运动会实现竞赛成绩和精神文明双丰收。我市列入信息惠民国家试点城市。平安鹰潭、法治鹰潭建设加快推进，信访维稳、应急处置、安全生产、食品药品安全监管进一步加强，社会保持和谐稳定。

　　（七）政府自身建设进一步增强。深入开展“连心、强基、模范”工程和“四进三改两覆盖”活动，党的群众路线教育实践活动取得明显成效，161项意见建议全部整改结束。办复人大代表议案建议65件、政协提案120件，代表、委员满意率100%。完善政府工作规则，强化政府部门绩效考核和行政问责，电子政务综合办公平台试运行。全面落实党风廉政建设责任制和中央八项规定，开展整治“小金库”、“三公”经费、公务用车、办公用房等专项治理，加大审计监察和腐败案件查处的工作力度，全面推行政府法律顾问制度，政风行风建设取得新成效。

　　与此同时，统计、档案方志、外事侨务、气象服务、防灾减灾、民兵预备役、国防动员、拥军优属、人民防空、妇女儿童、老龄、残疾人等工作取得了新的成绩。

　　各位代表，成绩和进步来之不易。这些成绩的取得，是省委、省政府和市委正确领导的结果，是市人大依法监督、市政协民主监督的结果，是全市人民锐意进取、共同努力的结果。在此，我代表市人民政府，向全市广大干部群众，向各位人大代表、政协委员、各民主党派、工商联、无党派和社会各界人士，向驻鹰单位、人民解放军、武警官兵、公安民警和离退休老同志，向所有关心、支持、参与鹰潭经济社会发展的各界人士，表示衷心的感谢并致以崇高的敬意！

　　回顾过去一年的工作，我们清醒地看到，我市经济社会发展还存在不少困难和问题，主要表现在：经济总量不大，产业结构不优，重大项目支撑乏力，发展升级的压力仍然不小；中心城区辐射带动力不强，县域经济总体实力有待提升，统筹城乡发展的责任仍然重大；公共服务供给与民生改善需求存在差距，社会建设和管理的任务仍然繁重；转变政府职能、运用市场化手段推动发展的能力需要进一步提升，发展环境还需优化，建设法治型、服务型政府的要求仍然迫切。对此，我们一定认真对待，切实加以解决。

　　二、2015年工作安排

　　2015年是全面深化改革的关键之年，是全面推进依法治国的开局之年，也是全面完成“十二五”规划的收官之年。适应经济发展新常态，培育经济增长新动力，做好今年经济社会发展各项工作，最根本的是要始终保持发展升级的战略定力，坚决贯彻落实省委十六字方针，按照市委十六字总体要求，坚持把稳增长作为首要任务，持之以恒抓产业、抓项目、抓投入，做大总量，提升质量，助推发展升级；最重要的是要始终保持改革开放的创新定力，坚持市场化改革方向，突出问题导向，创新开放思路，把改革创新贯穿于各个领域、各个环节，进一步激发市场主体活力，增强发展动力；最核心的是要始终保持民生优先的施政定力，坚持把群众利益摆到最高位置，做到眼前利益与长远利益相统一，多思民生之盼，多谋民生之利，多解民生之忧，以民生福祉的不断增进，凝心聚力促发展；最关键的是要始终保持攻坚破难的实干定力，以“钉钉子”精神，一年一年干下去，一件一件抓落实，一步一个脚印向前进，使我们的发展更有活力、产业更具实力、城乡更富魅力。

　　今年政府工作的总体要求是：全面贯彻党的十八大、十八届三中、四中全会和习近平总书记系列重要讲话精神，认真落实省委十三届七次、八次、九次、十次全会精神，遵循“发展升级、小康提速、绿色崛起、实干兴赣”十六字方针，按照“主攻项目、决战‘三区’，凸现特色、实现跨越”总体要求，坚持稳中求进工作总基调，主动适应经济发展新常态，全力推进“8+1”重点工作，更加注重改革创新，更加注重转型升级，更加注重城乡统筹，更加注重民生改善，更加注重生态文明，奋力迈出打造世界铜都、中华道都、田园新城新步伐，为建设富裕秀美宜居和谐鹰潭、全面建成小康社会奠定坚实基础。

　　今年全市经济社会发展主要预期目标是：生产总值增长9%左右，财政总收入增长9%，一般公共预算收入增长9%，规模以上工业增加值增长11%，固定资产投资增长16%，社会消费品零售总额增长13%，城镇居民人均可支配收入增长10%，农村居民人均可支配收入增长10%，人口自然增长率控制在7.8％。以内，居民消费价格总水平涨幅控制在3%左右，完成省下达的节能减排计划任务。

　　实现上述目标，今年重点抓好以下九个方面的工作：

　　（一）着力推进工业强市，形成集群发展新态势。

　　坚持以“1+6”产业体系为方向，以沿320国道产业集聚升级带为主战场，深入实施工业强市战略，力争规模以上工业企业实现主营业务收入3600亿元。

　　1.加速铜产业转型升级。围绕建设世界铜都的战略目标，加快铜产业发展要素集聚，进一步延伸铜产业链条，努力打造“七位一体”的产业体系。做大铜精深加工，力促胜华集团22万吨铜杆、众鑫成铜业2万吨铜基复合线材等8个项目开工建设，红旗铜业12万吨、江冶实业1.8万吨高性能铜材等10个项目建成投产，力争全年铜精深加工和终端应用产品比重提高10个百分点。做活铜贸易市场，依托铜期货交割仓库和中江国际商品交易中心，健全完善铜贸易发展措施，大力开展铜贸易招商，加快形成集展示、交易、采购、仓储、配套服务于一体的交易平台，力争铜现货贸易交易量增长50%以上。做旺铜原料市场，以列入全省关检“三个一”试点为契机，积极争取废六类进口转关转检正常化和废铜绿色通道，加快进口废物原料拆解、循环经济技术研发与孵化中心等项目建设。做优铜研发检测平台，加快建设国家铜及铜产品质量监督检验中心，整合铜产业技术平台，积极申报铜基新材料战略性新兴产业区域集聚发展国家试点，增强铜产业科技创新能力。

　　2.壮大特色产业集群。按照“依托龙头带动一批、立足优势培育一批、招大引强扩展一批”的要求，加快推进水工、节能照明、硫磷化工、大健康等十大特色产业基地建设。狠抓工业项目建设，力争养元“六个核桃”20万吨饮品、百炼化工8.8万吨氟化新材料等10个项目年内投产见效，力促吉智食品、英弗五金等10个重大项目开工建设，加快黄金产业园、博兰得光伏小微逆变器等10个项目建设，增强工业发展后劲。

　　3.强化工业发展保障。推进沿320国道产业集聚升级带建设，实施工业园区提升计划，着力打造富有活力、可持续的“经济走廊”。创新体制机制，建立高新区市场化运作机制，推进工业园区扩区调区，力争龙岗工业园创建省级产业园。完善园区配套，着力完善与产业集群相配套的金融、物流、市场服务体系，加快小微企业孵化基地、科技孵化园、总部经济大楼等项目建设，确保电镀集控区投入运营，新增标准厂房5万平方米；大力实施“新市民”工程，推动园区向城市功能区转型。破解要素瓶颈，积极创新投融资模式，对竞争性领域的产业，政府由支持具体项目改为设立工业发展基金；用好“财园信贷通”等政策措施，规范运营市中小企业信用担保公司；加强企业用工帮扶，抓好职业技能培训，探索建立对外劳务合作机制。

　　（二）着力推进新型城镇化，构筑全域鹰潭新格局。

　　坚持以列入国家新型城镇化综合试点城市和全省“多规合一”试点市为契机，以信江新区为市域中心，全力实施“1+3+10”城镇发展模式，努力形成“四区同城、十镇联动、产城融合、智慧共享”的城镇化发展新格局。

　　1.突出中心城区带动。实施产城融合工程，出台扶持政策，积极引导总部经济和公共服务机构进驻中央商务区，大力推进天虹商场、鹰潭大厦等大型商业综合体建设，建成市民广场步行街，完善新区功能配套，使信江新区成为宜居宜业的核心区。实施棚户区改造工程，推进老一中、盐业公司、朱埠村、上下桂村等棚户区和城中村改造，科学布局社区文教卫体老等公共服务设施，打造市民“15分钟生活圈”。实施景观提升工程，突出信江两岸道路景观和慢行功能，加快鹰西大道滨江沿线和白露河景观提升改造工程、信江国家湿地公园、信江植物园、鹰西湿地公园等建设，营造“碧水绕城、绿树融城”的生态宜居环境。实施道路畅通工程，加快新区路网四期、环城东路延伸等16条城区主次干道建设，推动中心城区交通大贯通；加速鹰北公路客运综合枢纽站和公交枢纽站建设，争取鹰西客运站、鹰南公交枢纽站年底建成运营。实施便民服务工程，加快信息惠民国家试点城市建设，改造完善主城区农贸市场、垃圾中转站等市政配套设施，加快开放式群众性运动场、老城区公共停车场、供水供电供气管网、加气（油）站等项目建设，优化城区公交线路，构建网格化、标准化、数字化城市管理格局，进一步提升城市宜居水平。

　　2.构建新型城镇化格局。着力推进同城发展，编制完成鹰潭市域总体规划（2014—2030），加快320、206国道拓宽改造步伐，力争206国道改造年内全面竣工；开工建设余信贵大桥，启动信江南路延伸段、余信贵大道贵溪段和余江段建设，全力推进鹰贵、鹰余一体化发展。着力推进县城建设，认真落实好促进县域经济发展20条措施，加快县城新区拓展和老城改造步伐，推动贵溪市现代工业新城、余江县产城一体化示范试点区建设，尽快繁荣发展月湖新城。着力推进镇村联动，用好城乡建设用地增减挂钩和扩权强镇政策，加快推进十强中心镇建设，完成187个自然村点的秀美乡村建设任务。

　　3.健全城乡一体机制。围绕推进农业转移人口市民化，进一步推进户籍制度改革，统一城乡户口登记制度，调整户口迁移政策，力争城镇化率提高1.5个百分点。围绕公共服务一体化，探索建立政府、企业、个人“三位一体”成本分担机制，抓紧完善与户籍制度相配套政策，稳步推进教育、就业、养老、医疗卫生、住房保障等城镇基本公共服务覆盖全部常住人口。围绕投融资体制多元化，按照“政府主导、社会参与、市场运作”的原则，加强政府投资项目管理，鼓励社会资本通过土地运作、项目融资、特许经营等方式参与新型城镇化建设，逐步构建投资主体多元化、投资方式多样化、项目建设市场化的投融资体制新格局。

　　（三）着力推进服务业发展，强化转型升级新支撑。

　　充分发挥服务业对一、二次产业的重要促进作用，坚持一二三产业融合发展，促进现代服务业发展提速、比重提高、水平提升，力争服务业增加值突破200亿元。

　　1.加速发展旅游业。树立全域旅游理念，整合旅游资源，推动龙虎山主景区、中心城区、白鹤湖示范区三大板块旅游功能融合、要素互补，着力打造全国著名的休闲旅游目的地。主攻核心景区，加快龙虎山景区产品开发和功能配套步伐，力争地质花卉公园、逍遥城、圣井山漂流、梦幻乐园等项目建成投入使用，启动天师林、农业生态园、大上清宫二期、景区路网等项目。加大“寻梦龙虎山”品牌推广力度，提升演出的知名度和影响力，使其成为新的旅游消费热点。推进全域旅游，挖掘道文化、铜文化等文化积淀，整合北极阁、眼镜博物馆、古玩一条街、黄蜡石市场等旅游资源，完善旅游宾馆等服务配套，规划建设慢行旅游通道，加快城景一体步伐。推进白鹤湖示范区旅游休闲功能开发，建成白鹤湖大道，启动滨湖生态游憩区、苗木花卉景观带等项目，整合周坊、画桥红色旅游资源，着力形成“南有龙虎山、北有白鹤湖”发展格局。强化营销管理，以创建智慧景区为契机，推进景区运行机制市场化、服务标准化建设，依托高铁开通百趟旅游专列，提升整体营销策划和宣传水平，力争全市接待国内游客增长38%，旅游总收入增长45%。

　　2.繁荣发展商贸物流业。按照“城内大商贸、城郊大市场、城乡大流通”的布局，加快鹰南现代物流园、鹰西国际商贸园建设，推进万寿宫商城、农机大市场及大型商业综合体等项目建设，力争林安商贸物流园一期、胜华机电五金商贸城二期、大三江电商物流港一期等项目竣工。强化政策引导，严格划行归市，规范手拉手汽车城、浙商联盟建材家居城、国际眼镜城等专业市场管理，尽快形成市场辐射效应。完善货运信息平台、物流公共服务平台等配套设施。健全电商交易网络平台，推进赣东北电商谷、中部电子商务国际贸易中心等项目建设，促进电商、网购、快递等融合发展，形成区域性电子商务产业集群。推进“万村千乡市场”工程，加快实施农村商品配送中心、农贸市场改造升级。

　　3.创新发展新兴服务业。大力发展普惠金融，积极引进股份制银行、城商行设立分支机构，确保小微企业和“三农”贷款增速增量不低于各项贷款平均水平。推动“三农”政策性保险扩面。支持开展民间借贷登记管理机构试点工作，积极防范金融风险。大力发展文化产业，加快“四馆合一”、文化创意产业园及特色街、铜文化产业园、余江雕刻产业园建设，做大做强黄蜡石市场。鼓励社会力量进入健康养老服务领域，加快市社会福利养老中心建设，提升改造15个农村敬老院，力争建成48个社区居家养老服务中心。积极发展家政社区、信息咨询等新兴业态，引导房地产业健康发展。

　　（四）着力推进“三农”工作，构建现代农业新体系。

　　按照“稳粮增收、提质增效、创新驱动”的总要求，不断挖掘新潜力、培育新优势、拓展新空间，促进农业增效、农民增收、农村增色。

　　1.加快现代农业发展。抓好示范化引导，以白鹤湖列入国家级现代农业示范区为契机，加大涉农资金整合和统筹使用力度，加快现代农业核心示范区基础设施建设，大力引进落户一批优质现代农业项目。抓好特色化产业，积极培育壮大蔬菜、果业、茶叶、休闲农业等八大特色产业，做大做强油茶、早熟梨、毛竹、葡萄等7个万亩生产基地，推进林下经济“12233”工程建设。抓好标准化生产，新增省级以上农产品商标名牌和“三品一标”20个，建成畜禽标准化规模养殖场15个。

　　2.创新经营体制机制。完成农村土地承包经营权确权登记颁证工作，探索农村土地经营权抵押贷款制度，推进农村集体产权制度改革和余江农村宅基地制度改革试点，深化供销合作社综合改革，不断激活农业农村发展活力。加快农村产权流转交易市场建设，新增流转土地5万亩。深入开展“财政惠农信贷通”试点，大力培育新型农业经营主体，力争新增市级以上农业龙头企业20家以上，销售收入达80亿元以上，新增家庭农场110家、农民专业合作社460家。

　　3.增强综合生产能力。落实各项强农惠农富农政策。做好永久基本农田划定工作，新建高标准农田12万亩，确保粮食总产69万吨以上。深入实施第二轮全国小农水重点县建设，完成小型水利工程管理体制改革试点，完成72座小（二）型病险水库除险加固工程，加快东川河改造、月湖童家联圩等项目建设，力争新增和恢复灌溉面积1.2万亩。推进城市公交向农村延伸，改造农村公路360公里。完善农技推广、农产品检测体系，提高农业生产安全保障能力。

　　（五）着力推进改革攻坚，增添发展升级新动力。

　　坚持问题倒逼改革，致力把已出台的重大改革举措落实好，新推出一批既惠当前又利长远的高质量改革举措，把更多的改革红利有效转化为发展新动力。

　　1.继续深化行政体制改革。认真做好行政审批事项“接、放、管”工作，深入推进“三单一网”建设，制定公布责任清单和负面清单，加快全市电子政务综合办公平台和网上审批系统建设，实现所有事项全流程、并联式网上公开审批。稳妥推进事业单位分类改革。推进不动产统一登记制度改革，完成不动产登记职责整合。推动执法权重心下移，加大跨部门、跨行业综合执法力度，推进市场监管综合执法。加强中介服务管理，规范和培育中介服务市场，探索建立“中介超市”。

　　2.继续完善现代市场体系。深化工商登记制度改革，减少工商登记前置行政许可事项，全面实行“先照后证”登记制，进一步放宽市场准入，大力培育市场主体。建立涉企收费清单管理制度，规范减少涉企收费，减轻企业负担。进一步创新重点领域投融资机制，大力推行政府与社会资本合作（PPP）等多种投融资模式。进一步完善国有资产管理体制，不断健全现代企业管理制度，实行国有企业上缴国有资本收益制度，推行市级投融资公司企业化运营机制，提高国有资产运营效益，做大做强国有资产。推进价格领域改革。启动“信用鹰潭”建设工程，完善小微企业信用体系建设。

　　3.继续深化财税体制改革。建立统一规范的部门预算体系，所有使用财政资金的部门除涉密信息外一律公开预决算。清理、规范、整合财政专项资金，最大限度发挥使用效益。规范政府举债融资，将政府债务纳入预算管理。推进税制改革，进一步扩大“营改增”范围。加强结余结转资金管理，规范非税收入管理和财税优惠政策。进一步规范实施好29个政府购买服务试点项目，逐步扩大试点范围。

　　（六）着力推进开放升级，拓展内生发展新空间。

　　坚定不移实施大开放主战略，在更大范围、更宽领域、更高层次深化区域开放合作交流，充分激发创新创业创造活力，进一步增强经济发展的后劲和支撑。

　　1.扩大开放水平。突出招大引强，按照“大项目—产业链—产业群—产业基地”的发展方向，策应全省重大项目招商引资三年攻坚计划，强化境外海外招商，探索实施咨询公司、专家代理、招商顾问等招商模式，紧盯行业龙头企业，力争全年引进省外5000万元以上项目资金276亿元，实际利用外资2.37亿美元。推动外贸转型升级，培育壮大重点出口企业，力争实现外贸出口9.4亿美元。

　　2.加强“三争”工作。围绕对接国家“七大工程包”，高位跟进赣南等原中央苏区振兴发展、赣东北扩大开放合作等重大战略，编制完成“十三五”规划，建立覆盖全市统一的项目储备库。积极论证包装一批投资额大、牵动力强的重大项目，做细做实项目前期，努力提高项目申报的质量和效益，争取获得更多项目、资金和用地指标，力争鹰潭机场、华能核电、花桥水库等项目纳入国家和省计划盘子，确保争取上级资金增长20%以上。

　　3.推进创新创业。更加注重科技协同创新，加大人才培养引进力度，发挥研发平台创新驱动能力，新增省级研发平台2个、市级研发平台5个，实现高新技术产业增加值50亿元、增长20%。推进知识产权入园强企“十百千万”工程，实施质量兴市战略。全力支持本土人才大胆创业、在外鹰商回归创业，营造“引智兴鹰、创业兴鹰”的浓厚氛围。

　　（七）着力推进生态建设，走出绿色崛起新路子。

　　围绕创建国家森林城市，始终坚持在保护中发展、在发展中保护，严守生态红线，构建生态文明，努力在全省创建生态文明先行示范区中主动作为、创先争优。

　　1.加强生态环境保护。推进“净空”行动，加强大气污染防治，强化建筑施工现场标准化管理，推进公交车、出租车等天然气利用，启动PM2.5监测，空气质量保持国家二级标准以上。推进“净水”行动，大力实施水生态文明建设试点，加强信江流域和集中式饮用水水源地环境保护，加大洪五湖等中小型水库水质治理力度，推进海利化工、麒麟化工搬迁，确保城南、信江新区、龙虎山污水处理厂竣工投运。推进“净土”行动，加大农村人居环境整治力度，推广“户分类、村收集、镇转运、县处理”为主的城乡一体垃圾处理模式，扎实推进重金属污染治理和畜禽养殖等农业面源污染综合防治，开工建设生活垃圾焚烧发电项目。

　　2.推进资源节约集约利用。大力实施工业园区循环化、生态化改造，加快推进“城市矿产”和资源综合利用“双百工程”示范基地及潢溪省级循环经济基地建设。抓好工业、建筑、交通和公共机构节能，推进国家可再生能源建筑应用示范城市建设。大力推进光伏和风力等新能源项目建设。加强低丘缓坡地、未利用地综合开发利用，推进“旱地改水田”工作，确保耕地占补平衡。深入开展园区再造工程和闲置土地清理专项行动，加快消化批而未供土地。巩固农村建房“三清理一整治”专项行动成果。

　　3.创新生态文明体制机制。严把项目环保准入关，确保建设项目“三同时”执行率100%。推进重点企业环境污染强制责任保险试点工作。推广环境治理特许经营、合同能源管理、污染第三方治理和各类节能环保产品。加强林地红线保护管理，推进造林绿化“四大工程”建设，加快阳际峰自然保护区一级项目建设，完成造林绿化4.62万亩，力争森林覆盖率达58.2%。

　　（八）着力推进民生改善，开创社会事业新局面。

　　按照“保基本、兜底线、促公平、可持续”的原则，大力推进以保障和改善民生为重点的社会建设，加快完善基本公共服务体系，努力提升人民群众的幸福感和满意度。

　　1.办好民生实事。在全面完成省下达民生工程任务的基础上，围绕人民群众最关心、最直接、最现实的利益问题，全力做好就业创业、社会保障、教育公平、医疗卫生、食品安全、公益文化、保障性住房、交通出行、宜居鹰潭、法治鹰潭等十大惠民工程75件实事，确保全年民生支出占财政总支出的比重达60%以上。结合机关事业单位养老保险制度改革，完善工资制度，提高基本工资标准并建立正常调整机制。强化资金和质量监管，完善长效机制，确保民生工程发挥持久效益，让老百姓更多更好地分享改革发展成果。

　　2.发展社会事业。统筹城乡义务教育资源均衡配置，推进农村义务教育学校标准化建设和恒大九年一贯制学校、新二中等新改扩项目；整合职业教育资源，加快推进鹰潭职业技术学院迁建工程；深化教育领域综合改革，加快全省教育信息化试点建设，探索优质教育托管模式。强化公共卫生服务体系建设，建成市人民医院信江新院，加快市县公立医院改革和基层医疗卫生机构综合改革，鼓励扶持民营医院做大做强，实现村级卫生计生服务室标准化建设全覆盖。依法加强食品药品安全监管工作，建立覆盖生产、流通、消费全过程的信用信息平台。做好全国、省、市劳动模范评选表彰工作。完成第二次地名普查试点工作。大力发展群众体育和竞技体育。

　　3.创新社会治理。深入推进法治鹰潭建设，健全和创新普法体制机制，努力形成办事依法、遇事找法、解决问题用法、化解矛盾靠法的法治环境。依法推进社会治安综合治理，创新社会治安防控体系，强化社会面管控“四项建设”，加强社区规范化建设和网格化管理，发展社会组织，推动社会建设和管理重心下移。依法预防化解社会矛盾，建立律师参与信访接待和信访疑难案件第三方评查制度，推进信访省内终结制度，依法处理“以闹取利”的违法犯罪行为。加强网络监管和舆论引导，净化网络环境。建立健全事故防控及安全生产责任体系，完善应急处理机制，防范和遏制重特大安全事故。

　　同时，进一步做好统计、档案方志、外事侨务、涉台事务、气象和老龄工作，加强国防教育、人防民防、民兵预备役建设，支持各民主党派、工商联、知识分子联谊会和工会、共青团、妇联等人民团体的工作。

　　（九）着力推进自身建设，树立法治政府新形象。

　　党的十八届四中全会对政府自身建设提出了新的更高要求。我们将紧紧围绕法治政府建设，坚持在法治轨道上开展工作，创新治理方式，加快建设职能科学、权责法定、执法严明、公开公正、廉洁高效、守法诚信的法治政府。

　　1.强化法治意识，做到依法治市、依法执政、依法行政。健全领导干部学法守法用法考评机制，不断强化法治思维，提高依法行政能力。健全依法决策机制，完善重大行政决策法定程序，建立重大决策合法性审查机制、重大决策终身责任追究制度及责任倒查机制。依法规范行政行为，理顺事权，厘清责任，推进决策、执行、管理、服务、结果公开。坚持严格规范公正文明执法，严格执行行政裁量权基准制度，全面落实重大行政行为备案审查制度。自觉接受人大法律监督、政协民主监督和司法监督，虚心接受群众监督和舆论监督，切实加强监察、审计等内部监督，认真办理人大代表建议和政协提案。

　　2.强化实干意识，做到主动作为、敢于作为、善于作为。建立政府工作协调、反馈、督导机制，健全政府部门目标责任绩效考核机制，突出过程监管，严格督促检查，进一步加快政府工作节奏，提高工作效率。建设诚信政府，严格履行向社会作出的承诺，把经济社会发展目标落实情况和为群众办实事情况作为评价政府诚信程度的重要内容。优化发展环境，严格责任追究，坚决遏制不作为、乱作为、中梗阻等行为。

　　3.强化廉政意识，做到干部清正、政府清廉、政治清明。巩固和拓展党的群众路线教育实践活动成果，认真贯彻党风廉政建设责任制，全面落实“两个责任”，严格遵守中央八项规定，坚决反对“四风”，全面开展廉政风险防控，严格建设工程招投标、财政转移支付等公共资源管理权力的监管，以零容忍态度惩治腐败。牢固树立过“紧日子”思想，严控“三公”经费支出，完善清理“吃空饷”长效机制，积极稳妥推进公务用车改革，全面规范办公用房、公务接待，营造风清气正的良好政治生态。

　　各位代表！方向凝聚力量，实干催生巨变。让我们紧密团结在以习近平同志为总书记的党中央周围，在省委省政府和市委的坚强领导下，进一步解放思想、开拓进取，主动适应新常态，积极谋求新动力，努力展现新作为，为建设富裕秀美宜居和谐鹰潭作出新的更大贡献！
